# Caveolae Contribute to the Apoptosis Resistance Induced by the α_1A_-Adrenoceptor in Androgen-Independent Prostate Cancer Cells

**DOI:** 10.1371/journal.pone.0007068

**Published:** 2009-09-18

**Authors:** Maria Katsogiannou, Charbel El Boustany, Florian Gackiere, Philippe Delcourt, Anne Athias, Pascal Mariot, Etienne Dewailly, Nathalie Jouy, Christophe Lamaze, Gabriel Bidaux, Brigitte Mauroy, Natalia Prevarskaya, Christian Slomianny

**Affiliations:** 1 Inserm U800, Université Lille 1 Sciences et Technologies, Villeneuve d'Ascq, France; 2 Laboratoire de Physiologie Cellulaire, Université Lille 1 Sciences et Technologies, Villeneuve d'Ascq, France; 3 Lipidomique-IFR100, Hôpital du Bocage, Dijon, France; 4 IFR 114, IMPRT, Institut de Recherche sur le Cancer de Lille, Lille, France; 5 Institut Curie, Centre de Recherche, Laboratoire Trafic, Signalisation et Ciblage Intracellulaires, Paris, France; 6 CNRS, UMR144, Paris, France; Baylor College of Medicine, United States of America

## Abstract

**Background:**

During androgen ablation prostate cancer cells' growth and survival become independent of normal regulatory mechanisms. These androgen-independent cells acquire the remarkable ability to adapt to the surrounding microenvironment whose factors, such as neurotransmitters, influence their survival. Although findings are becoming evident about the expression of α_1A_-adrenoceptors in prostate cancer epithelial cells, their exact functional role in androgen-independent cells has yet to be established. Previous work has demonstrated that membrane lipid rafts associated with key signalling proteins mediate growth and survival signalling pathways in prostate cancer cells.

**Methodology/Principal Findings:**

In order to analyze the membrane topology of the α_1A_-adrenoceptor we explored its presence by a biochemical approach in purified detergent resistant membrane fractions of the androgen-independent prostate cancer cell line DU145. Electron microscopy observations demonstrated the colocalisation of the α_1A_-adrenoceptor with caveolin-1, the major protein component of caveolae. In addition, we showed that agonist stimulation of the α_1A_-adrenoceptor induced resistance to thapsigargin-induced apoptosis and that caveolin-1 was necessary for this process. Further, immunohistofluorescence revealed the relation between high levels of α_1A_-adrenoceptor and caveolin-1 expression with advanced stage prostate cancer. We also show by immunoblotting that the TG-induced apoptosis resistance described in DU145 cells is mediated by extracellular signal-regulated kinases (ERK).

**Conclusions/Significance:**

In conclusion, we propose that α_1A_-adrenoceptor stimulation in androgen-independent prostate cancer cells *via* caveolae constitutes one of the mechanisms contributing to their protection from TG-induced apoptosis.

## Introduction

Prostate cancer is one of the most common forms of cancer in men and the second cause of cancer death in industrialized countries [1]. Various factors such as androgens and growth factors regulate epithelial cell proliferation and apoptosis in the normal prostate and early-stage prostate cancer (PCa). Androgen ablation is currently the leading therapy used to block the growth of androgen-dependent cancer cells. However PCa cells' proliferation and survival often become independent of regulatory mechanisms leading to a hormone-refractory disease [2] for which there is currently no successful therapy. Androgen-independent PCa cells have the remarkable ability to adapt to the surrounding microenvironment whose influence on intracellular survival pathways remains subject to debate [3]. Indeed, PCa cells are in contact with various factors such as hormones, growth factors and neurotransmitters which are thought to influence the physiology of these cells. Among others, interest has been shown for the endogenous catecholamines norepinephrine and epinephrine. In fact, the subepithelial stroma of the prostate is particularly rich in autonomic nerves and α_1_-adrenoceptors (α_1_-AR). The α_1A_-AR subtype, in particular, is found in smooth muscle cells but its expression has also been described in epithelial cells [4], [5]. The α_1A_-AR is a member of the superfamily of G-protein coupled receptors (GPCR) mediating actions of the previously mentioned catecholamines in a variety of cells [6].

α_1_-AR antagonists are already used for the clinical treatment of benign prostate hyperplasia (BPH) [7], where their therapeutic benefit is attributed to a direct action on α_1_-AR present in prostate smooth muscle cells [8]. However, several studies have provided evidence on additional effects of α_1_-AR antagonists such as doxazosin on long-term BPH treatment. These agents have been demonstrated to inhibit prostate growth by inducing apoptosis in stromal and epithelial cells and are emerging as potential therapeutic regimens for the prevention and treatment of androgen-independent PCa [9], [10], [11]. In addition, previous studies from co-workers on human prostate cancer epithelial (hPCE) cells and the androgen-dependent prostate cancer cell line LNCaP showed that phenylephrine (PHE), an α_1A_-AR agonist, stimulates their proliferation [12], [13]. Despite these promising findings, the functional role of α_1A_-AR in androgen-independent PCa cells has yet to be established.

It has been described that the signalling and trafficking of several GPCR are regulated by specialized plasma membrane domains known as lipid rafts [14]. Moreover, recent data on cardiomyocytes have shown that α_1_-AR as well as the molecules involved in its signal transduction pathway are accumulated in caveolae, a subclass of membrane microdomains [15], [16]. Caveolae are 50–100 nm flask-shaped plasma membrane invaginations, characterized on one hand by high contents of cholesterol and glycosphingolipids and on the other hand by the presence of caveolin-1 (cav-1), the major constitutive protein of 20–25 kD [17]. Interestingly, cav-1 has been associated with many diseases such as atherosclerosis and Alzheimer's disease [18], [19]. Regarding its role in cancer, it has been established that PCa is associated with increased cav-1 expression [20]. In fact, this protein has been identified as a marker associated with PCa progression and hormone-refractory disease, playing a determinant role in the androgen-independence of PCa cells [21]. By its association with specific receptors and enzymes on the plasma membrane, cav-1 can be a direct mediator of survival, growth and metastasis signals in PCa cells [22].

To date, not much is known about the mechanisms underlying neurotransmitters involvement in the survival of PCa cells, let-alone the role of α_1A_-AR in androgen-independent epithelial cells and PCa progression. In this regard, it is tempting to link the presence of α_1A_-AR and cav-1 and to hypothesize that the α_1A_-AR could mediate *via* caveolae its functional effects on growth or survival of advanced stage PCa cells.

The objective of our work was therefore to explore the role of the α_1A_-AR in androgen-independent PCa cells. Here, we investigate the presence of α_1A_-AR in caveolae of DU145 cells, an androgen-independent PCa cell line derived from brain metastasis. We analyze the consequence of α_1A_-AR stimulation by PHE on the receptor and cav-1 membrane distribution as well as its effect on the lipid composition of membrane raft fractions purified from DU145 cells. Furthermore, we describe the effect of PHE in the apoptosis resistance of these cells through activation of ERK and our results strongly imply the involvement of caveolae in this signalling pathway. Finally, by immunohistofluorescence and RT-PCR, we observe a positive correlation of α_1A_-AR and cav-1 expression and advanced stage PCa. The presence of α_1A_-AR-rich caveolae could therefore contribute to the generalized apoptosis resistance characterizing androgen-independent prostatic tissue.

## Methods

### Cell culture

The androgen-independent human prostate cancer cell line DU145, obtained from the American Type Culture Collection, was maintained in culture in RPMI 1640 medium (Life Technologies, Inc) supplemented with 10% (v/v) FCS (Seromed, Poly-Labo, Strasbourg, France), 5 mM L-glutamine and 2 mM kanamycin (Sigma). Cells were routinely grown in 50 ml flasks (Nunc, PolyLabo, Strasbourg, France) at 37°C in a humidified 5% CO_2_-95% air atmosphere. These cells constitute a suitable model for brain metastatic cancer cells present in hormone-refractory prostate cancer.

### Cell transfections and generation of cav-1 knock-down DU145 cell line

Two ready-to-use siRNAs against α_1A_-AR (siADR1A, Eurogentec) were transiently transfected (50 nM) with HiPerfect Transfection Reagent (Qiagen) in DU145 cells. Control siRNA (siCTL) experiments were performed by transfecting siRNA against Luciferase.

siLuciferase (siCTL) : 5′-CUUACGCUGAGUACUUCGA-3′. siADR1A-1 (mix of): 5′-CAGGAAAGAUGCAGAGGA-3′ and 5′-UUCCUCUGCAUCUUUCCU-3′. siADR1A-2 (mix of): 5′-GCGUCUACGUGGUGGCCA-3′ and 5′-UUGGCCACCACGUAGACG-3′.

Stable cell lines were obtained by transfecting DU145 cells with a Psuper vector encoding short hairpin RNA (shRNA) against cav-1 (sequence: CTGGAATAAGTTCAAATTCTT 2121 3′utr) (DUshcav-1) (or empty Psuper vector for control cell line, DUshCTL) using Nucleofector, as recommended by the manufacturer (Amaxa GmbH, Köln, Germany). We transfected 2×10^6^ trypsin-treated DU145 cells with 3 µg of vector and then used the transfected cells to seed a 24 well plate (Nunc) at very low density. Clones underexpressing cav-1 were selected on the basis of their antibiotic resistance to puromycine and validated by Western Blot.

### Antibodies and Reagents

Primary antibodies used for immunofluorescence (IF) microscopy and immunoblotting (IB) were: (1∶100) rabbit anti-α_1A_ adrenoceptor (Santa Cruz Biotechnology), (1∶100) rabbit anti-caveolin-1 (Santa Cruz Biotechnology), (1∶50) mouse anti-caveolin-1 (BD transduction Laboratories), (1∶400) mouse anti-β-actin (Sigma), (1∶200) anti-calnexin (Chemicon, Millipore, Paris, France), (1∶1000) mouse anti-cytokeratin 18 (Chemicon, Millipore, Paris, France), (1∶1000) rabbit anti-PARP (Cell Signaling), (1∶100) mouse anti-bax (6A7) (Santa Cruz Biotechnology) recognizing an exposed epitope of bax in the activated conformation, (1∶1000) rabbit anti-procaspase 3 (UPSTATE), (1∶1000) anti-phospho-p44/42 MAP Kinase and (1∶1000) anti-p44/42 MAP Kinase (Cell Signaling). Secondary antibodies used for IB were: (1∶10000) horseradish peroxidase-linked anti-mouse or anti-rabbit (Chemicon). For IF, we used (1∶1000) Alexa Fluor® 488-labelled and (1∶2000) 546-labelled secondary antibodies (Molecular Probes).

Cells were treated with 10 µM L-Phenylephrine Hydrochloride (PHE), 1 µM prazosin (PRA) and 10 µM PD98059 for three days (renewed every 24 h) and 10 µM Thapsigargin (TG) for 48 hours in RPMI medium. Short term PHE treatments were realized in a solution containing (mM): NaCl, 116; KCl, 5.6; CaCl_2_, 1.8; MgCl_2_, 1.2; NaHCO_3_, 5; NaH_2_PO_4_, 1; HEPES, 20; pH 7.3. All chemical products were provided by Sigma except TG (Alomone) and PD98059 (Calbiochem).

### Cell cycle analysis

Cells were grown in three 60-mm dishes per condition and drugs were applied as described above. After treatments, cells were trypsinized, harvested and resuspended in 0.2 ml sterile PBS. 1 ml of cold 70% ethanol was added onto cell suspensions while vortexing. Samples were centrifuged, washed in sterile PBS and then incubated with ribonuclease (2 µg/ml) for 15 min. Propidium iodide (25 µg/ml final in PBS-triton X-100 0.1%) was then added and allowed to incubate for an additional 30 min. DNA content was measured by exciting propidium iodide at 488 nm and measuring the emission at 520 nm (FL3) using a flow cytometer (Beckman coulter Epics XL4-MCL with Expo32 acquisition). Experiments were repeated four times.

### Western Blot

The cell culture medium was discarded and flasks were washed with iced PBS. Cellular proteins were then extracted using RIPA buffer [1% (v/v) Triton X-100, 1% (w/v) Na deoxycholate, 150 mM NaCl and 20 mM sodium or potassium phosphate, pH 7.2] with 5 mM EDTA and anti-proteases cocktail (P8340; Sigma) for 30 min on ice. After scraping, any insoluble material was removed by centrifugation at 30000×*g* for 10 min at 4 °C and the amount of protein was assessed by the BCA method (Pierce Chemical Company, Rockford, IL, U.S.A.). Equal amounts of proteins were subjected to SDS/PAGE (16% gels). Finally, the proteins were transferred on to nitrocellulose membranes using a semi-dry electro-blotter (Bio-Rad). After 1 h saturation in 5% non-fat milk (or 5% BSA (bovine serum albumin) only for anti-phospho-p44/42 MAPK), membranes were incubated overnight with diluted primary antibodies (see “Antibodies and Reagents”). The membranes were then washed (3×10 min) with a TNT buffer (15 mM Tris/HCl, pH 8, 140 mM NaCl and 0.05% Tween 20) and treated with the corresponding horseradish peroxidase-linked secondary antibodies (anti-mouse or anti-rabbit, Pierce), (1∶10000) diluted in TNT/milk (or TNT/BSA) for 1 h at room temperature. After several washes in TNT buffer, the membranes were processed for chemiluminescent detection using the Super Signal West Dura chemiluminescent substrate (Pierce) according to the manufacturer's instructions. The membranes were finally exposed to X-Omat AR films (Eastman Kodak Company, Rochester, NY, U.S.A.). The intensity of the signals was evaluated by densitometry and semi-quantified using the ratio for each sample between the intensity of protein of interest divided by the actin or calnexin intensity. Each experiment was repeated at least twice.

### Isolation of Detergent Resistant Membranes (DRM) and Dot blot

Cells were rinsed, scrapped, centrifuged and the supernatant was removed. The pellet was resuspended with isolation buffer B (according to Axis-Shield S33 Application Sheet) and homogenized with a Dounce homogenizer (Kontes) of 15 µm pestle clearance then centrifuged for 10 min at 1000×*g*. The supernatant corresponding to crude lipid raft extract was isolated and 0.2% Triton X-100 was added. A discontinuous 5-step Optiprep® (Axis-Shield PoC AS) gradient was formed with buffer D (buffer B+1% Triton X-100) (w/v) in order to obtain 1.66 ml of 35%; 2.5 ml of 20%; 2.5 ml of 15%; 2.5 ml 10%; 0.83 ml of 5%. Crude lipid raft extract was made dense with sucrose (230 mg for 0.5 ml of lysate) and laid at the interface between gradients 35% and 20%. Ultracentrifugation at 165400×*g* for 4 h at 4°C in 50 Ti rotor (Beckman Coulter) was followed by collection from the bottom up of 1.1 ml fractions. 1.1 ml of 20% (v/v) TCA (Trichloroacetic Acid) was added to each fraction in order to precipitate proteins. Tubes kept on ice for 20 min were centrifuged at 10000×*g* at 4°C for 10 min. The precipitate was washed with methanol, centrifuged then re-suspended in 4% (v/v) SDS. Since samples were not highly concentrated in proteins and detection by classical western blot was difficult to obtain, samples were loaded on nitrocellulose membrane in a 96-well Dot-Blot unit according to the manufacturer's instructions (Bio-Rad). Briefly, the ten fractions were spotted onto nitrocellulose membrane in series. When dry, the membrane was incubated in blocking solution for 1 h (TNT/milk) then hybridized with the desired antibodies following classical IB procedure as described above (see “Western Blot”). Precautions were accordingly taken in keeping some important factors constant for the treated and non-treated conditions. The cell density used for the experiments was constant and when cold detergent extraction was carried out both the concentration of the detergent and the ratio of cell number/detergent concentration were the same. Each well was loaded with 25 µl from each fraction. This standardized method was repeated at least three times and was used to evaluate changes in the partitioning of a target molecule in DRM, avoiding false estimations when based on the relative amounts with regard to all other molecules present.

### Quantification of phosphatidylcholine (PC) and sphingomyelin (SM)

Lipids were extracted according to the method of Folch et al. [23]. Dimyristoylphosphatidylcholine (DMPC Sigma), dimyristoylphosphatidylserine (DMPS Avanti Polar Lipids), lauroylsphingomyelin (LSM Avanti Polar Lipids) were used as internal standards. Phospholipid analysis was performed on a Hypersil Si 2×200 mm column (Agilent Technologies, Massy, France) following protocol [24]. Positive ESI-MS was performed on a MSD 1100 Mass Spectrometer (Agilent Technologies). The orifice voltage was set at 120 V, the capillary voltage at 3.5 kV, the drying gas (Nitrogen) flow at 8 l/min and scan range from m/z 400 to 950. Integrated peak were as from Extracted Ion Chromatograms (EIC) for m/z = 700 to 950 at the retention time (RT) of PC, PS, SM were divided by the EIC for m/z = 679 at the RT of DMPC, m/z = 681 at the RT of DMPS or m/z = 647 at the RT of LSM. Levels were determined by comparison of this ratio with a standard curve of known amounts of phosphatidylcholine and sphingomyelin.

### Quantification of Cholesterol

Extraction and saponification were performed according to the previously described procedure [25] with some modifications. Quantification of cholesterol was performed using a HP6890 Gas Chromatograph equipped with an HP7683 Injector and a HP5973 Mass Selective Detector (Agilent Technologies). Chromatography was performed using a HP-5MS fused silica capillary column (30 m×0.25 mm inner diameter, 0.25 µm film thickness, Agilent Technologies). A selected ion-monitoring program was used for mass spectrometry. The ions used for analysis (m/z) were as follows: epicoprostanol, 370; cholesterol, 368. Calibration curves were obtained by analyzing standards prepared from authentic standards (Steraloids) and extracted with the method used for samples.

The above quantification protocols for lipids and cholesterol were repeated three times for three individual experiments in non-treated (control) and treated DU145 cells with PHE for 10 min. As the amounts of lipids and cholesterol quantified in each fraction (ng/ml or µg/ml) varied slightly among experiments, results were normalised by determining mean values for the three experiments represented by the percentage of lipid and cholesterol in each fraction in control conditions compared to PHE-treated conditions.

### Plasma Membrane “Rip Off”

This method was based on a published protocol [26]. Cells were grown on glass coverslips. Formvar®-coated copper grids (according to [27]) were coated with polylysine. Coverslips were placed cell side down onto the grids and a slight pressure was exerted using a rubber bung then coverslips were turned over and the grids were carefully detached then fixed in 8% (v/v) PFA (paraformaldehyde). After washing in PBS and saturation in PBS/2.5 mM, glycine/donkey serum immunolabelling was carried out according to standard techniques, using primary antibodies followed by species-specific anti-IgG gold conjugates of 6 nm, 12 nm and 18 nm. Finally, grids were contrasted and prepared for observation on a Hitachi H-600 transmission electron microscope (magnification ×20000) at 75 kV.

### Ultrastructural Microscopy

For transmission electron microscopy, Cells were fixed in 2.5% glutaraldehyde prepared in 0.1 M cacodylate buffer and post-fixed in 1% osmium tetroxide in the same buffer. After acetonitril dehydration, the pellet was embedded in Epon. Serial thin sections (90 nm) were cut using a Reichert Ultracut E ultramicrotome and collected on 150 mesh hexagonal barred copper grids. After staining with 2% uranyl acetate prepared in 50% ethanol and incubation with a lead citrate solution, sections were observed on a Hitachi H-600 transmission electron microscope.

### Indirect Immunofluorescence

Resection BPH specimens from human prostate frozen in liquid nitrogen-cooled isopentane and kept in “Tissue-Tek®” at −80°C before 10 µm sections were prepared at −20°C with a cryostat, mounted on glass slides and proceeded to IF study. Normal and cancerous prostate tissues were supplied by tissue-array slides (SuperBioChips Laboratories), deparaffinized according to manufacturer's indications (Cliniscience) before preparation for IF according to [28]. Briefly, all slides were blocked during 30 min with PBS/1.2% (v/v) gelatin then incubated with desired primary antibodies for 1 h at 37°C then secondary antibodies (1 h, 37°C). Imaging was performed using Zeiss LSM 510 confocal head (Carl Zeiss) connected to a Zeiss Axiovert 200 M microscope with a ×40 oil-immersion objective lens (numerical aperture 1.45). Slides were scanned using an argon ion laser and a helium/neon ion laser. All scanning parameters were kept constant throughout the different experiments. AIM 3.2 confocal microscope software (Carl Zeiss) was used for data acquisition and analysis.

### RT-PCR

Reverse transcription-PCR was carried out as previously described [29]. The 178 pb α_1A_-AR isoform 1 amplicon was amplified with 5′-AGACCAATCCTCCTGTACCAC-3′ (forward) and 5′-CTCTGCATCTTTCATGTCCTAG-3′ (reverse). The 220 pb cav-1 amplicon was amplified with 5′-AGTGCTCCTGTTCTCCCTTC-3′ (forward) and 5′-CTTGTCGATGGCTTCCTTCAC-3′ (reverse) (Eurogentec). The 236 pb GAPDH amplicon internal control was amplified with 5′-TTCACCACCATGGAGAAGGC-3′ (forward) and 5′-GGCATGGACTGTGGTCATGA-3′ (reverse). The 241 pb cytokeratin 18 amplicon was amplified with 5′-TGAGTCAGAGCTGGCACAGA-3′ (forward) and 5′-TGGTGTCATTGGTCTCAGACA-3′ (reverse). Finally, the 210 pb cytokeratin 14 amplicon was amplified with 5′-TGCGAGATGGAGCAGCAGAA-3′ (forward) and 5′-TGCCATCGTGCACATCCATGA-3′ (reverse). α_1A_-AR, cav-1, cytokeratin 14 and 18 mRNA expressions were validated by gel density analysis by using GAPDH mRNA as internal control.

### Human Tissue specimens

Human BPH biopsies were obtained from consenting patients following the local ethical considerations. All experiments involving patient tissues were carried out under approval number ‘CP 01/33’, issued by the ‘Comité Consultatif de Protection des Personnes dans la Recherche Biomédicale de Lille'.

### Statistical analysis

Results were expressed as the mean±SEM. Statistical analysis was performed using unpaired *t* tests (for comparing two groups) or analysis of variance tests followed by Dunnett (for multiple control *versus* test comparisons). Differences were considered significant when *p*<0.05 (*), *p* <0.01 (**) and *p* <0.001 (***).

## Results

### α_1A_-AR is associated with cav-1 at the DU145 cell surface

**Figure 1:**
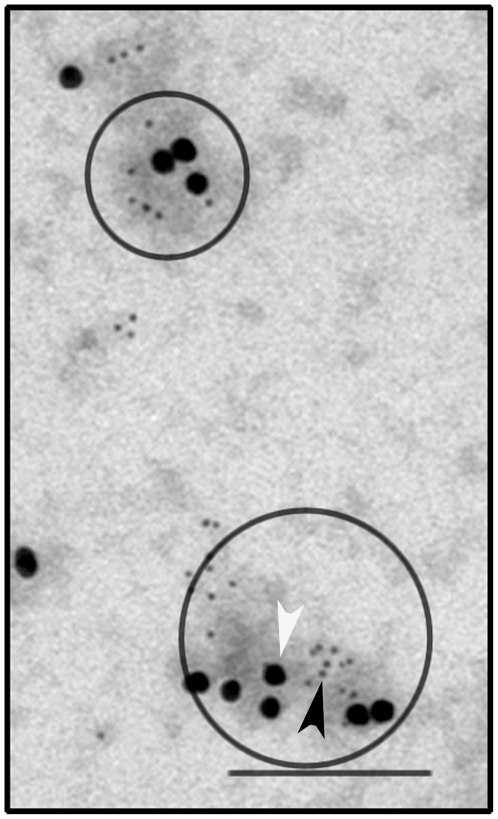
α_1A_-AR and cav-1 colocalization in DU145 cell surface. Plasma membranes were “ripped-off” as described in the “Methods” section and incubated with anti-cav-1 and anti-α_1A_-AR antibodies. Secondary antibodies coupled with 6 nm and 18 nm gold particles were used against anti-α_1A_-AR (black arrowhead) and anti-cav-1 (white arrowhead) respectively. Grids were then processed for electron microscopy observation. Colocalization of both proteins is indicated by circles. Bar, 200 nm.

Previous studies have described a direct interaction of α_1A_-AR with cav-1 [15]. Moreover, α_1A_-AR signalling is known to localize in caveolae [14], [30]. To explore the membrane topology of α_1A_-AR, we used the “rip-off” technique developed in adherent cells allowing the isolation and observation by electron microscopy of large areas of the plasma membrane [26]. α_1A_-AR immunogold staining (6 nm dots, black arrowhead) colocalizes with cav-1 (18 nm dots, white arrowhead) in plasma membrane microdomains of about 50–100 nm (Figure 1).

### Plasma membrane protein redistribution and lipid composition alterations in DRM fractions induced by phenylephrine

**Figure 2:**
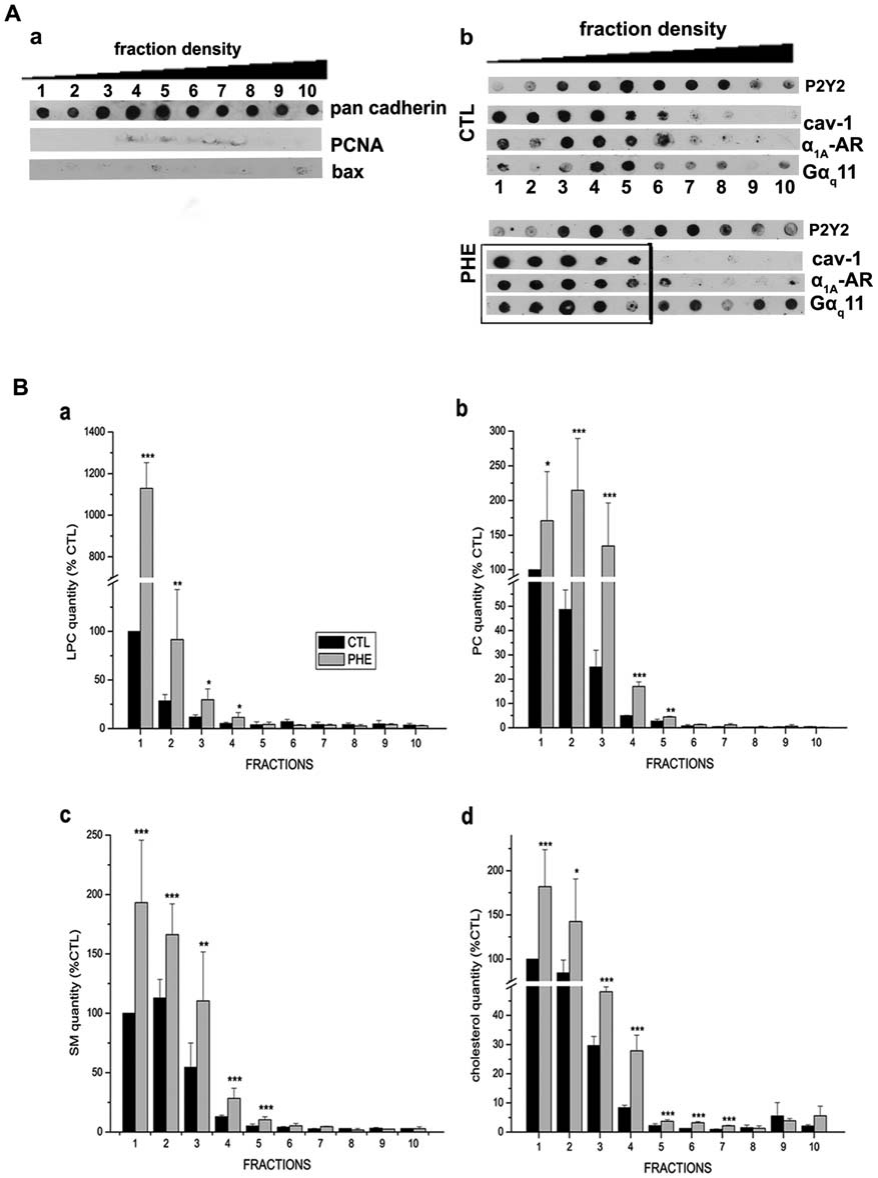
Protein and lipid redistributions after agonist stimulation of the α_1A_-AR by phenylephrine. (A) DRM fractions of increasing density (from fraction 1 to 10) obtained as indicated in “Methods” section and analyzed by Dot blot. (a) Pan cadherin was used as a positive control confirming that the fractions obtained correspond to plasma membrane. PCNA (proliferation cell nuclear antigen) and Bax were used as negative controls, their absence confirming the non-contamination of DRM fractions by intracellular proteins known not to be associated with rafts. (b) Dot blot of the DRM fraction collection in control condition (CTL) and 10 min treatment with 10 µM phenylephrine (PHE). Purinergic receptor P2Y2 was used as a negative control for the specificity of the effect of phenylephrine on protein redistribution in fractions. Black rectangle marks the shift of α_1A_-AR, cav-1 and Gα_q11_ protein to lighter density fractions. (B) Lipid composition of the 10 fractions was quantified as described in the “Methods” section in control (black column) and PHE treated cells (grey columns) for (a) lysophosphatidylcholine (LPC), (b) phosphatidylcholine (PC), (c) sphingomyelin (SM), (d) cholesterol. Error bars represent SEM calculated from three independent experiments. Statistical analysis used the *t* test; *, *p*<0.05, **, *p* <0.01 and ***, *p* <0.001.

Analysis of plasma membrane microdomains, caveolae or lipid raft composition typically begins with detergent solubilization of whole cells followed by density gradient centrifugation and recovery of DRM from light fractions of the gradient. We investigated the association of α_1A_-AR with purified DRM from DU145 whole cell membrane extracts on an OptiPrep® density gradient. For the characterisation of DRM fractions, the presence of a specific plasma membrane marker pan cadherin was first assessed as a positive control (Figure 2A, a). Its presence in all 10 purified fractions demonstrates that the isolated DRM fractions correspond to plasma membrane regions. In addition, the nuclear protein PCNA (proliferation cell nuclear antigen) and the mitochondrial protein bax were used as negative controls as they are known not to associate with plasma membrane. Their absence in DRM fractions validates the absence of contamination by intracellular membranes (Figure 2A, a).

In order to determine whether previously defined microdomains are involved in the α_1A_-AR signalling pathway in DU145 cells, we examined α_1A_-AR stimulation by its specific agonist phenylephrine (PHE). Dot blot analysis of DRM fractions revealed distribution of cav-1 in low density gradient (5%–20%) fractions 1–6 and α_1A_-AR in fractions 1 and 3–6 in control (CTL) conditions. After 10 minutes treatment by 10 µM PHE cav-1 distribution was observed in low density gradient (5%–10%) fractions 1–5 and α_1A_-AR was now detected in all fractions 1–6 (including low density fraction 2) (Figure 2A, b, upper panel CTL; lower panel PHE). Importantly, Gα_q11_ (an α_1A_-AR effector) contents showed a shift from fractions 4–5 to fractions 1 to 5 after PHE treatment and remain present in higher density fractions, suggesting that receptor activation does not involve all Gα_q11_ proteins present at the membrane level. We also assessed the purinergic receptor P2Y2 used as a negative control for the specificity of modification in protein distribution induced by PHE. α_1A_-AR and P2Y2 are both GPCR sharing similar signalling pathways. P2Y2 is located in the same fractions whether DU145 cells were treated or not (Figure 2A, b, CTL and PHE), therefore demonstrating the specificity of the previously observed PHE-induced modified protein distribution.

Moreover, we were interested in the lipid composition of purified DRM fractions. Lipids known to be enriched in biological lipid rafts, such as lysophosphatidylcholine (LPC), sphingomyelin (SM), phosphatidylcholine (PC) and cholesterol [31], [32], were analyzed. Once again, we investigated possible alterations of the lipid composition of DRM fractions after PHE stimulation. Our observations show a significant increase in the quantities of these lipids mainly in low density gradient (5%–10%) fractions 1–5 as a result of PHE stimulation (Figure 2B, a, b, c, d). These results are in agreement with the well established fact that the high lipid content causes floating of lipid microdomains in low density fractions during centrifugation. Previously published data on lipid model systems show that the size of lipid rafts depends on the lipid membrane composition [33], [34]. The observed alterations in lipid compositions due to membrane reorganization may account for lighter density microdomains resulting in the redistribution of α_1A_-AR, cav-1 and Gαq_11_ observed within DRM fractions.

### Protein and lipid reorganization induced by phenylephrine is associated with cav-1-rich membrane clustering

**Figure 3:**
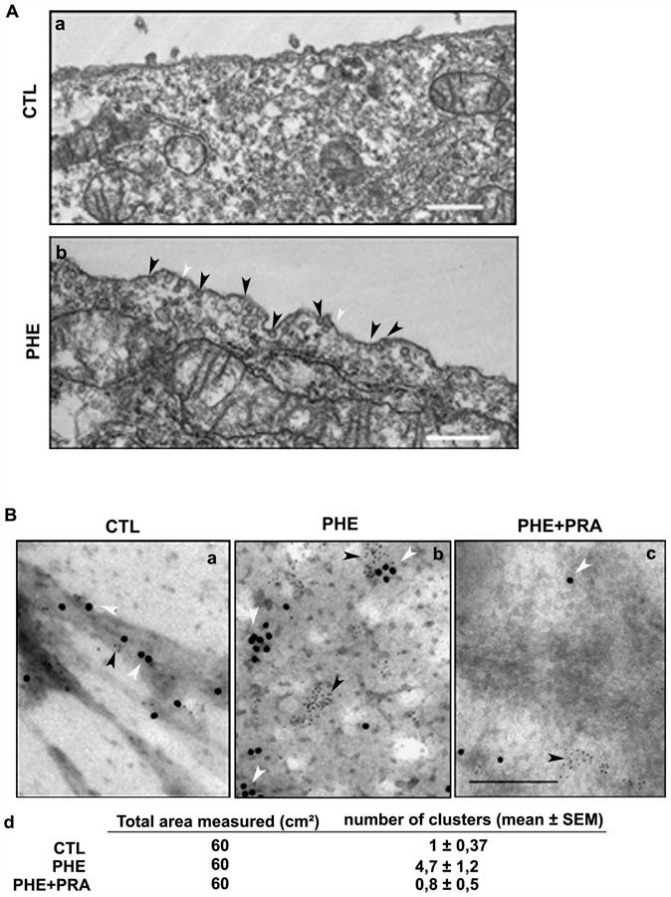
Cell surface modification and caveolin-1 mobilization following α_1A_-AR stimulation. (A) Representative electron micrograph of DU145 cells in (a) non-treated condition (b) after 10 min treatment by 10 µM PHE. Caveolae and caveolin containing vesicles of 50–80 nm diameter are dense in electrons are indicated by black arrowheads. White arrowheads indicate complex shaped caveolae. Bar, 500 nm. (B) Representative electron micrograph of plasma membrane “ripped-off” from (a) non-treated cells, (b) treated for 10 min with 10 µM PHE alone and (c) PHE in the presence of 1 µM PRA. 6 nm gold particles represent anti-α_1A_-AR labelling (black arrowheads) and 12 nm particles represent anti-cav-1 (white arrowheads). Bar, 200 nm. (d) The area of randomly selected negatives of electron micrographs (ten/condition) was measured at a magnification of 20000. The number of cav-1 and α_1A_-adrenoceptor-containing clusters (minimum 3 gold particles for each protein) was counted (mean ± SEM).

It is thought that upon extracellular stimulus, the plasma membrane is prepared for the formation of more stabilized domains and molecular clusters with enhanced size and lifetime such as caveolae [35], [36]. In order to understand the involvement of caveolae in the α_1A_-AR signalling, we explored the effect of PHE stimulation on the DU145 cell surface morphology (Figure 3A). Cells treated for 10 min with 10 µM PHE present numerous surface invaginations corresponding to caveolae (Figure 3A, b) as compared to non-treated cells (Figure 3A, a). Caveolae are evident as circular profiles with uniform shape and 50–80 nm diameter. In this representative electron micrograph caveolae are present as single pits (Figure 3A, b, black arrowheads) and sometimes in more complex arrangements interconnected with cytoplasmic caveolar profiles (Figure 3A, b, white arrowheads).

Caveolae are formed by the polymerisation of caveolins leading to the clustering and invagination of existing cholesterol-sphingolipid rich domains in the cell plasma membrane [36]. We investigated the membrane distribution of cav-1 and α_1A_-AR in control conditions and after α_1A_-AR stimulation (Figure 3B). The same surface areas of preparations (ten/condition) were compared in control and treated conditions and the mean number of clusters was calculated (Figure 3B, d). Here, representative electron micrographs for each condition are presented. We observed a greater number of cav-1-rich domains in PHE treated cell surfaces (Figure 3B, b) as compared to control conditions (Figure 3B, a). Further, in order to test the specificity of PHE stimulation on cell surface distribution of cav-1 and α_1A_-AR, we used an α_1_-AR antagonist, prazosin (PRA) simultaneously with PHE (Figure 3B, c). Cav-1 was present in isolated units and not in clusters at the cell surface associated with α_1A_-AR. It should be noted that PRA alone did not affect membrane localization of either cav-1 or α_1A_-AR (data not shown). Our results propose that α_1A_-AR may associate with cav-1 in resting cells and upon receptor stimulation clustering of cav-1-rich units form α_1A_-AR-containing structural surface caveolae.

### Phenylephrine sensitizes DU145 cells' resistance to Thapsigargin-mediated apoptosis through the caspase-3 pathway in a cav-1 dependent manner

**Figure 4:**
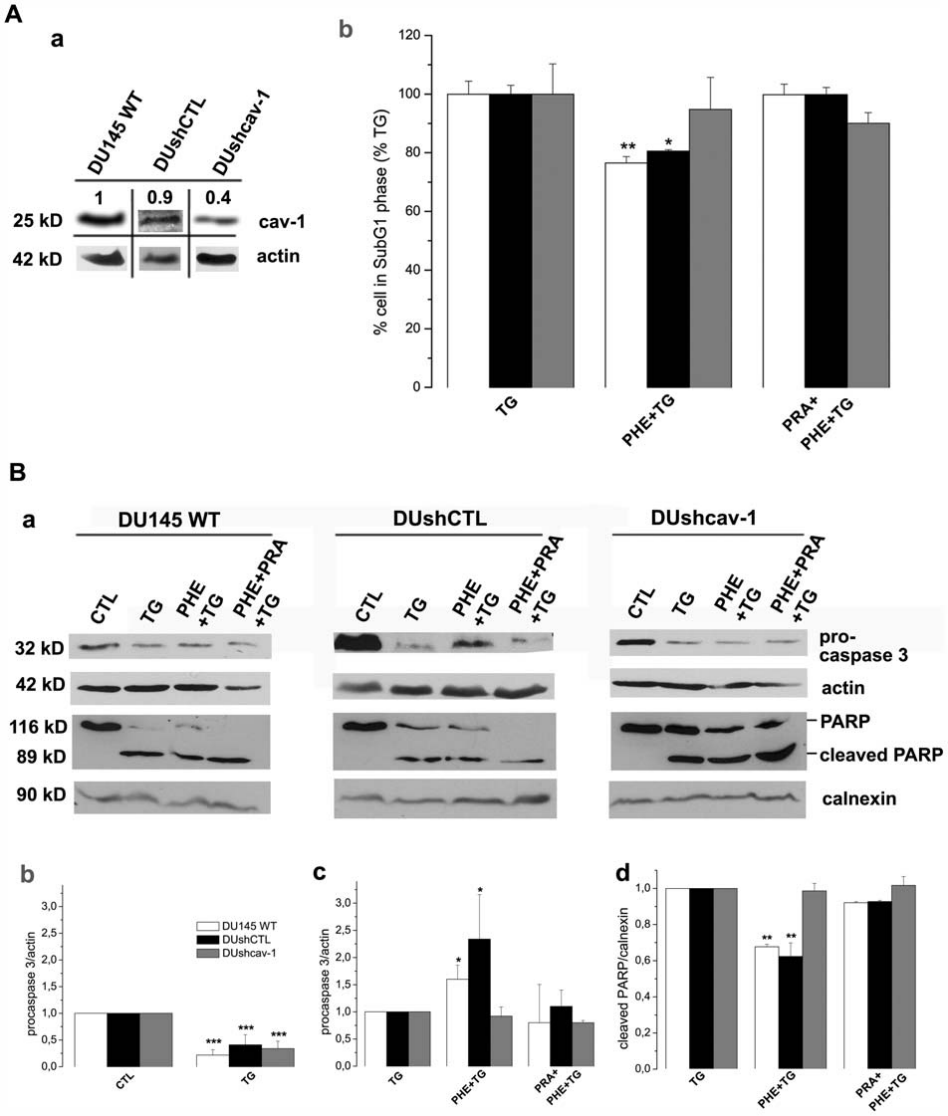
Apoptosis resistance of DU145 cells depends on caveolin-1 expression and is mediated by the caspase-3 pathway. (A)(a) Western blot showing the expression of caveolin-1 (cav-1) in DU145 cells stably transfected with Psuper plasmid expressing shRNA against cav-1 (DUshcav-1) and with empty Psuper plasmid (DUshCTL). Images are grouped from different lanes of the same gel and numbers above gels indicate relative expression assessed on actin expression. (b) Cells were treated during 48 hrs with 10 µM Thapsigargin (TG) for induction of apoptosis, preceded by a 3-day renewable treatment with 10 µM phenylephrine alone (PHE+TG) or simultaneously with prazosin (PRA+PHE+TG), then analyzed by flow cytometry of propidium iodide-stained nuclei. Experiments were carried out in DU145 non-transfected cells (DU145 WT, white columns), DUshCTL cells (black columns) and DUshcav-1 cells (grey columns). Error bars represent SEM calculated from four independent experiments. Statistical analysis used *t* test; *, *p*<0.05, **, P<0.01. (B)(a) Expression levels of pro-caspase 3 and PARP (full-length and cleaved fragment) in DU145 WT, DUshCTL and DUshcav-1, after treatments indicated above were determined by western blot analysis and analyzed by scanning densitometry using actin immunoblotting (for procaspase-3) and calnexin (for cleaved PARP) as internal controls. (b, c, d) Average densitometries are represented by histograms for non-transfected (white columns), control shRNA (black columns) and caveolin-1 shRNA expressing cells (grey columns). Plots are the average cumulative data (mean ± SEM) of three experiments. Statistical analysis used the *t* test; *, *p*<0.05, **, *p* <0.01 and ***, *p*<0.001.

We first quantified cav-1 in DU145 non-transfected cells (DU145 WT) and in stably transfected DU145 cells with either an empty plasmid (DUshCTL) or a plasmid expressing shRNA against cav-1 (Figure 4A, a). These cav-1 knockdown cells express 60% less cav-1 than non-transfected or shCTL cells. The use of these cells allowed us to investigate the involvement of caveolae in the α_1A_-AR signalling as it is further explained.

In order to study the role of α_1A_-AR signalling in the survival of DU145 cells, we induced cell apoptosis by a 48 h treatment with 10 µM thapsigargin (TG), a known inhibitor of sarcoplasmic/endoplasmic reticulum calcium ATPase (SERCA). As a consequence of inhibiting SERCA, TG induces a substantial depletion of calcium stores, creating perturbations in cellular calcium known as reticular stress coupled with the ability to engage mitochondrial-dependent apoptotic pathways [37]. In the present study, apoptosis was measured as an increased number of cells in SubG1 phase of the cell cycle [38]. It should be noted that the percentage of apoptotic cells in non-treated conditions was close to 0% and a 48 h TG treatment induced an increase of cells in SubG1 phase to about 20% (Supporting Information, Figure S1). In order to assess the effect of PHE on the TG-induced apoptosis, here we considered TG-treated cells as 100% in SubG1 phase (Figure 4A, b) and different treatments were therefore compared to TG-treated conditions.

A three-day pre-treatment by 10 µM PHE prior to TG treatment induced a significant 25% decrease in the number of DU145 WT cells (white columns) in SubG1 phase as well as a 20% decrease in DUshCTL (black columns) (Figure 4A, b). In order to demonstrate the α_1_-AR specificity of the apoptosis resistance induced by PHE, PRA was simultaneously used with PHE. Indeed, the resistance to TG-induced apoptosis was abolished when 1 µM PRA was added to the pretreatment solution (Figure 4A, b, black and white columns). It should be noted that PHE or PRA alone have no effect on cell cycle phases (Supporting Information, Figure S1, D). We then sought to investigate the involvement of caveolae in this phenomenon. We therefore disrupted caveolae structure by underexpressing cav-1. Analysis of SubG1 phase revealed that in cav-1 knockdown cells PHE pre-treatment did not promote resistance to TG-induced apoptosis (Figure 4A, b, grey columns). All these results were confirmed by the Hoechst staining (Supporting Information, Figure S2). The present findings strongly suggest the involvement of caveolae in the resistance to TG-induced apoptosis mediated by PHE in DU145 cells.

We then explored the expression of target proteins known to regulate mechanisms of apoptosis induction or resistance such as the caspases and the poly (ADP-ribose) polymerase (PARP) by western blot analysis (Figure 4B, a). Scanning densitometry allowed us to represent relative average densities of three different experiments by histograms (Figure 4B, b, c and d). We first assessed the expression of the 32 kD protein procaspase 3 in DU145 WT, DUshCTL and DUshcav-1 cells (Figure 4B, a). Our results in DU145 WT and DUshCTL cells showed a decrease of procaspase 3 after TG treatment due to its cleavage to active caspase 3 (Figure 4B, a), reflecting the induction of apoptosis and indicated respectively by a 60–80% decrease in intensity represented by the average ratios (Figure 4B, b, white and black columns). The TG-induced decrease of procaspase 3 was reversed by 60% and 134% respectively when DU145 WT and DUshCTL cells were pre-treated with 10 µM PHE, indicating decreased apoptosis (Figure 4B, a and c, white and black columns). Further, when 1 µM PRA was used simultaneously with PHE, procaspase 3 levels were similar to TG alone pointing out the antagonistic action of PRA to PHE (Figure 4B, a and c, white and black columns). On the contrary, in DUshcav-1 cells, PHE alone or in the presence of PRA did not reverse the 66% decrease of procaspase 3 induced by TG (Figure 4B, a and c, grey columns).

PARP, a 116 kD nuclear poly (ADP-ribose) polymerase is one of the main cleavage targets of caspase-3 *in vivo* [39]. Cleavage separates the PARP amino-terminal DNA binding domain (24 kD) from the carboxy-terminal catalytic domain (89 kD) [40] and serves as a marker of cells undergoing apoptosis [41]. In all our cell lines, TG treatment induced cleavage of PARP (Figure 4B, a), revealed by an antibody which recognizes both the full-length 116 kD fragment as well as the 89 kD cleaved fragment (see the “Methods” section). A three-day treatment by 10 µM PHE preceding TG reduced the amount of cleaved PARP by 30% and 35% suggesting decreased apoptosis in DU145 and DushCTL cells respectively (Figure 4B, a and d, white and black columns). 1 µM PRA pretreatment opposed the PHE-induced decrease in PARP cleavage only in DU145 and DUshCTL cells (Figure 4B, a and d, white and black columns). In Dushcav-1 cells, PHE treatment with or without PRA had no effect in PARP cleavage as compared to TG alone (Figure 4B, a and d, grey columns) once again strongly suggesting the involvement of caveolae in this function of α_1_-AR.

### TG-induced apoptosis resistance in DU145 cells is also mediated by a Bcl-2 family protein in a cav-1 dependent manner

**Figure 5:**
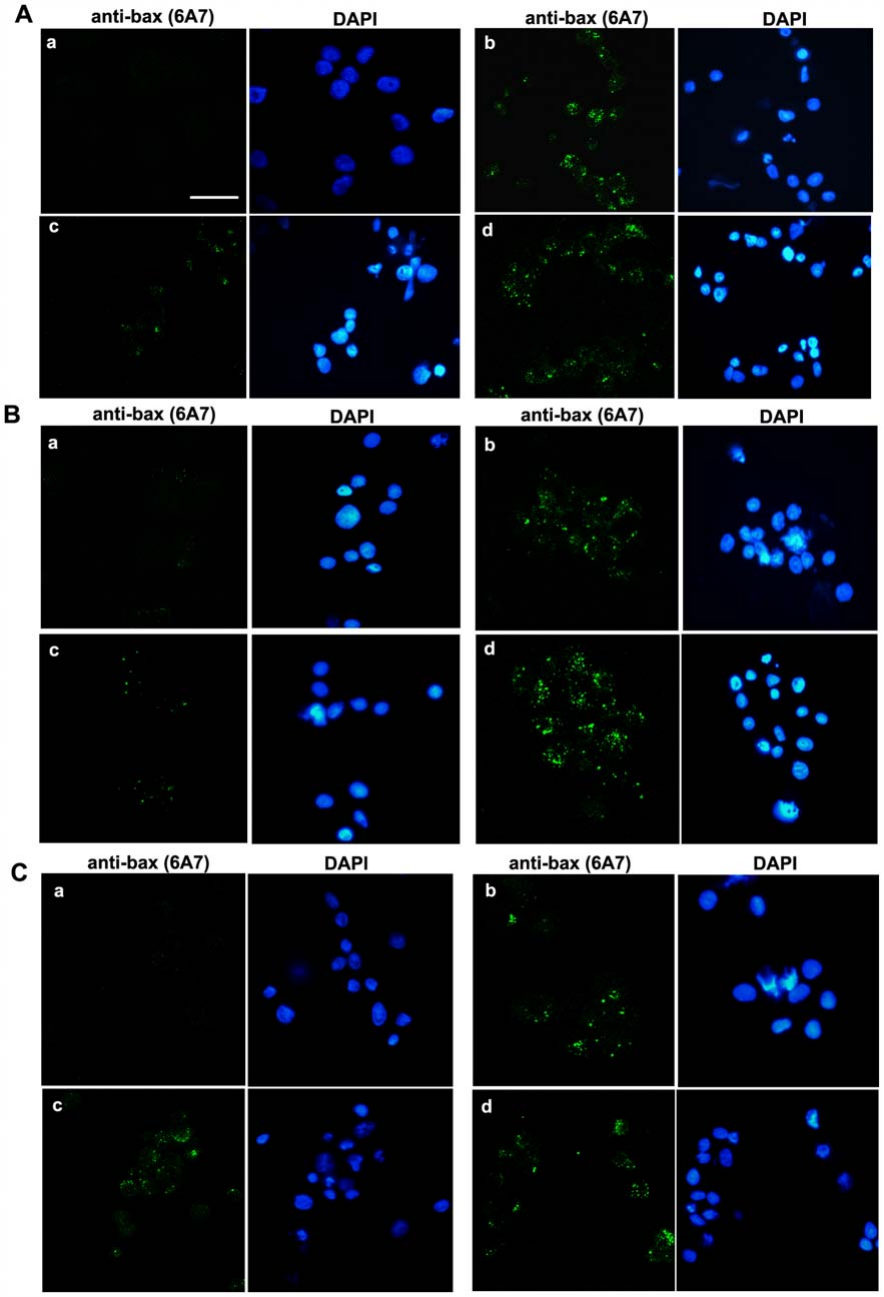
PHE enhances TG-induced apoptosis resistance in DU145 cells also *via* bax activation. Representative immunofluorescence of (A) DU145, (B) DushCTL and (C) Dushcav-1 cells in (a) non-treated conditions, (b) 10 µM TG for 48 h, (c) 10 µM PHE 3-day pretreatment (d) with the presence of 1 µM PRA, followed by 10 µM TG for 48 h showing the presence of activated Bax recognised by the anti-bax (6A7) antibody. The presence of cells in the observation field is indicated by DAPI-stained nuclei. Bar, 50 µm.

Bax is a 23 kD pro-apoptotic protein, member of the Bcl-2 family. The critical events in the activation process of Bax are: its translocation to mitochondria and its N-terminal conformational change closely coupled to mitochondrial membrane insertion and oligomerisation [42], [43]. The insertion of Bax into the mitochondrial outer membrane is closely associated with the release into the cytosol of several proteins such as cytochrome c and procaspase-3 which are essential to the execution of the apoptotic program [44]. We investigated Bax activation by immunofluorescence using an antibody which specifically recognizes the activated conformation of Bax (see “Methods” section). The figures shown here are representative of all slides observed. TG treatment of DU145 WT (Figure 5A, b), DUshCTL cells (Figure 5B, b) and Dushcav-1 cells (Figure 5C, b) induced an increase in activated-Bax staining as compared to non-treated conditions (Figure 5A, B and C, a). In DU145 and DushCTL cells, a three-day treatment by 10 µM PHE preceding TG reduced activated-Bax expression (Figure 5A and B, c) as compared to TG treatment alone (Figure 5A and B, b) suggesting decreased apoptosis in these conditions. Once more, 1 µM PRA pretreatment opposed the PHE-induced decrease in activated-Bax staining (Figure 5A and B, d). No significant consequence of PHE pretreatment (with or without PRA) in the resistance to TG-induced apoptosis was observed in DUshcav-1 cells (Figure 5C, b, c and d) once again strongly suggesting the involvement of caveolae in this function of α_1_-AR. DAPI-stained nuclei are indicative of the number of cells present in the observation field. The absence of apoptotic figures (chromatin condensation and nuclear fragmentation) in cells expressing activated Bax is expectable as Bax activation is an early-stage apoptotic process as opposed to nuclear fragmentation.

### Expression and localization variations of cav-1 and α_1A_-AR are associated with pathological alterations of human prostate tissue

**Figure 6:**
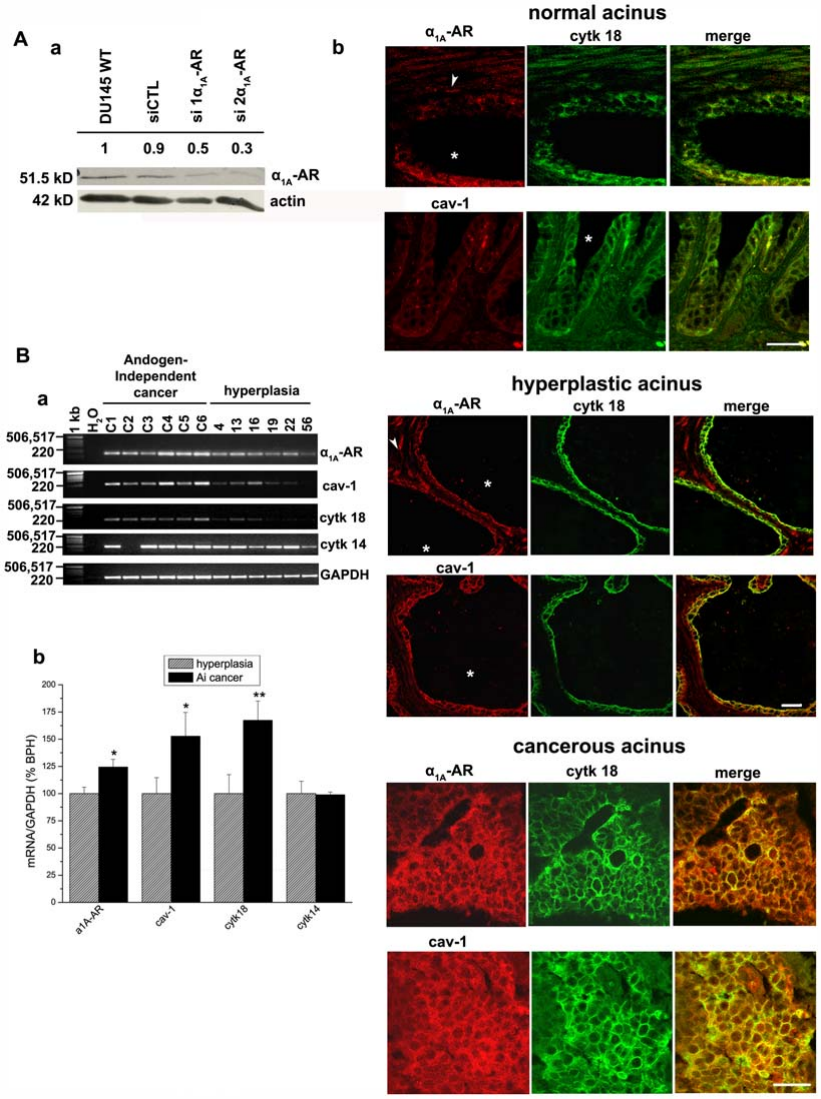
α_1A_-AR and cav-1 differential expressions in normal, cancerous and hyperplastic human prostate. (A)(a) Western blot showing the expression of α_1A_-AR in DU145 cells transfected with two siRNA against α_1A_-AR (siADR1A-1 and siADR1A-2) and control siRNA (siCTL). Numbers above gels indicate relative densitometry assessed on actin expression. (b) Representative immunofluorescence of normal prostate acinus showing the presence of the apical epithelial marker cytokeratin 18 (cytk 18, green) and α_1A_-AR or cav-1 fluorescence (red); Bar, 50 µm. Star indicates the acinus lumen and white arrowhead fibromuscular stroma. The middle left panel shows representative stage III cancerous acinus. Typically, cancerous apical epithelial cells detected with cytokeratin 18 (green) have completely invaded the lumen and express both α_1A_-AR and cav-1 (red); Bar, 50 µm. The middle right panel shows a section of a representative hyperplastic acinus where the lumen (star) is enlarged; Bar, 20 µm. (B)(a) Agarose gel showing the expression of α_1A_-AR (178 pb), cav-1 (220 pb), cytokeratin 18 (241 pb) and cytokeratin 14 (210 pb) amplicons in six different prostatic androgen-independent carcinoma tissues and six different benign hyperplasia tissues. A no-template control was also run with the PCR samples where cDNA was replaced with water (H_2_O). A 1-kilobase DNA ladder (*MW* (bp)) was used as a DNA size marker. GAPDH (236 pb) was used as an internal control. (b) Ratios of mean mRNA quantities of α_1A_-AR, cav-1, cytokeratin 18 (cytk 18) and 14 (cytk 14) as compared to GAPDH in androgen-independent (AI) cancer (black columns) and hyperplastic samples (grey hatched columns). Plots are the average cumulative data (mean ± SEM) of six samples by condition. Statistical analysis used the *t* test; *, *p*<0.05, **, *p*<0.01.

The α_1A_-AR is abundant in the fibromuscular tissue in normal and hyperplastic prostates [45], [46] however, its role in prostate epithelial cells is still not well defined. Moreover, cav-1 has been described to play an important role in the survival/growth of PCa cells contributing to their metastatic activities [47], [48]. To determine the association of α_1A_-AR and cav-1 expression with established features of PCa as well as BPH in comparison to normal prostate, we performed α_1A_-AR and cav-1 immunostaining on tissue microarrays containing specimen cores from 40 patients. Cytokeratin 18 immunostaining was used as a label of apical epithelial cells present in acini. Normal tissue regions from 9 of these patients were present on these slides. The figures shown here are representative of all samples observed. We first confirmed the specificity of the α_1A_-AR antibody by the use of two siRNA against α_1A_-AR (siADR1A-1 and siADR1A-2) (Figure 6A, a). The use of these siRNA induced a 40% and 60% decrease of α_1A_-AR as compared to DU145 cells transfected with the control siRNA. Previous studies revealed that cav-1 overexpression is associated with aggressive PCa [49] but little is known about α_1A_-AR expression in PCa progression. Here, we found that cav-1 and α_1A_-AR were strongly expressed in luminal, invasive epithelial cells in stage III PCa (Figure 6A, b, cancerous acinus). Furthermore, in hyperplastic tissues cav-1 and α_1A_-AR immunostaining was present in the simple lining of cytokeratin 18-positive-acinar epithelium and in the fibromuscular stroma (Figure 6A, b, hyperplastic acinus). The majority of the normal regions of the samples expressed low levels of cav-1 and α_1A_-AR in apical epithelial cells and the presence of the two proteins was mostly observed in the stroma (Figure 6A, b, normal acinus). Overall, these results demonstrated elevated expressions of cav-1 and α_1A_-AR in advanced PCa epithelial cells, more numerous and invasive in these conditions than in BPH.

Finally, we were interested in assessing the mRNA levels of α_1A_-AR and cav-1 in a series of cancerous and hyperplastic human prostate samples. We analyzed six PCa samples obtained from patients in hormone-refractory phase and characterized as androgen-independent and six BPH samples (Figure 6B, a). Relative quantification of α_1A_-AR and cav-1 mRNA levels as compared to GAPDH internal control was quite promising (Figure 6B, b). We found average mRNA levels of α_1A_-AR and cav-1 more elevated in androgen-independent PCa samples than in BPH (Figure 6B, b, black and hatched grey columns). Noticeably, cytokeratin 18 mRNA was more abundant in androgen-independent cancer samples than BPH, in agreement with our previous observations in immunofluorescence. Finally, we noticed that basal epithelial cells' marker cytokeratin 14 mRNA levels did not present any variations between androgen-independent prostate cancer and BPH (except for sample C2 for which no information is available to allow an explanation for its non-detection). Our data strongly suggest that α_1A_-AR may be associated with androgen-independent PCa epithelial cells and advanced stage PCa just like cav-1. Regardless of internal variability of hyperplastic and cancerous samples (related to factors such as patients' age, progression of disease, treatment) we underlined significant elevated mRNA levels of α_1A_-AR and cav-1 in androgen-independent cancer compared to BPH. We propose that their simultaneous expression could account in part for the α_1A_-AR signalling *via* caveolae in androgen-independent PCa epithelial cells.

### PHE protection from TG-induced apoptosis in DU145 cells involves activation of the ERK1/2 signalling pathway

**Figure 7:**
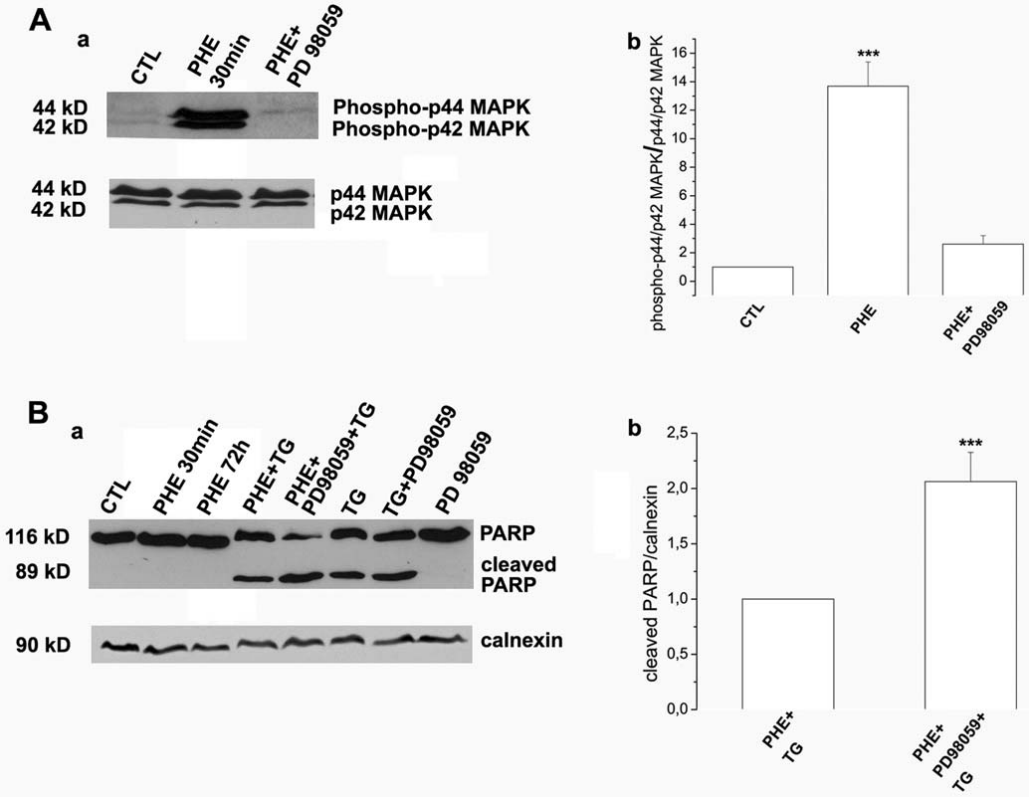
TG-induced apoptosis resistance in DU145 cells is mediated by ERK1/2. (A)(a) Western blot showing expression of phosphorylated p44/p42 MAPK (ERK1/2) in non-treated cells (CTL), 30 min treatment by 10 µM PHE (PHE 30 min) and simultaneous treatment by 10 µM PHE and 10 µM PD98059 (PHE+PD98059). (b) Expression of phospho-p44 and phospho-p42 MAPK is assessed on total p44/p42 MAPK in DU145 cells. (B)(a) Western blot showing expression of PARP full length fragment (116 kD) and cleaved PARP (89 kD) in the following treatment conditions: non-treated (CTL), 30 min or 72 h treatment by 10 µM PHE (PHE 30 min, PHE 72 h), pre-treatment by PHE alone or in the presence of 10 µM PD98059 followed by 48 h treatment by 10 µM TG (PHE+TG and PHE+PD98059+TG), 48 h treatment by 10 µM TG alone (TG) or preceded by 10 µM PD98059 pre-treatment (PD98059+TG) and 10 µM PD98059 treatment alone. (b) Expression of cleaved PARP is assessed on calnexin used as internal control for PHE+TG and PHE+PD98059+TG treatment conditions. Plots are the average cumulative data (mean ± SEM) of three experiments. Statistical analysis used the *t* test; ***, *p*<0.001.

It is now well established that ERKs are overexpressed in advanced stage prostate cancer and play a significant role in prostate tumorigenesis [50], [51]. This family of serine/threonine kinases is involved in transmitting signals regulating cell survival and growth [52]. Recently, is has been shown that the α_1B_-AR activates ERK in submandibular gland cells *via* a mechanism dependent of membrane microdomain integrity [53]. In the present study, we were interested in assessing PHE-induced activation of ERK1/2 and the potential involvement of this signalling pathway in the TG-induced apoptosis resistance described in DU145 cells. A 30 min treatment by 10 µM PHE induced activation by phosphorylation of ERK1/2 (p44/p42 MAPK) (Figure 7A, a and b). We used the specific inhibitor of ERK1/2 phosphorylation, 10 µM PD98059, which drastically decreased amount of activated ERK1/2 (Figure 7A, a and b). In order to demonstrate the involvement of ERK1/2 in the TG-induced apoptosis resistance in DU145 cells, we assessed PARP cleavage, indicating induction of apoptosis (Figure 7B). Interestingly, after simultaneous pretreatment of DU145 cells by 10 µM PHE and 10 µM PD98059 followed by 10 µM TG, we detected a 2-fold increase of cleaved PARP (89 kD) as compared to PHE alone followed by TG, strongly suggesting increased apoptosis in the presence of PD98059 (Figure 7B, a, lanes 4 and 5, b). As previously described (Figure 4B, a and d) TG alone induced a higher level of PARP cleavage as compared to PHE-pretreatment conditions (lanes 6 and 4). It should also be noted that PARP cleavage was similar in TG treatment conditions alone or in the presence of PD98059 and that PHE and/or PD98059 alone did not induce PARP cleavage (lanes 2, 3 and 8).

## Discussion

Various locally produced and circulating factors maintain prostate cancer growth by acting through cellular receptors. Increasing evidence supports the involvement of GPCR in neoplastic transformation of the prostate [54], [55]. Moreover, cancerous prostate expresses increased levels of GPCR and their ligands (for example bradykinin, endothelin and their corresponding receptors) [56], [57] suggesting that GPCR signalling may always be “switched on” therefore contributing to the initiation and progression of the disease.

A growing number of recent data has shown the involvement of α_1A_-AR in human prostate pathology [58], [59]. Further lines of evidence have demonstrated that α_1A_-AR and its effector proteins are differentially distributed in surface caveolae of cardiac cells [15], [16]. Caveolae and cav-1 have been described to play prominent roles in various human disease phenotypes including cancer (for reviews [18], [60]). Interestingly, the expression of cav-1 has been identified to be closely associated with PCa malignant progression and highly expressed in androgen-independent cells [61], [20], [49].

Despite the above findings, the precise surface localization of the α_1A_-AR in androgen-independent PCa epithelial cells and its functional role in the proliferation and survival of these cells remain unknown. The present study is the first to demonstrate the localization of α_1A_-AR in caveolae of the androgen-independent PCa epithelial cells DU145. Our results from the exploration of the lipid-protein contents of purified DRM from these cells were particularly revealing. Noticeably, we provide evidence of significant increase in DRM lipids upon agonist stimulation of the α_1A_-AR. We propose that this elevated content of raft-specific lipids consequently leads to alterations of DRM density as it is known that high lipid composition causes floating of membranes to lower density fractions after centrifugation [62]. Our results after dot blot analysis strongly suggest that α_1A_-AR and cav-1 content is accordingly distributed toward lower density fractions.

Furthermore, lipid composition alterations may be explained by coalescence of cav-1-containing membranes. In agreement with this suggestion, we observed a remarkable surface membrane reorganisation and formation of a great number of cav-1-rich invaginations following α_1A_-AR stimulation. It is well established that caveolae are formed by the polymerisation of caveolins, leading to the clustering and invagination of a subset of sphingolipid/cholesterol-rich membrane domains [36]. The trafficking of intracellular caveolin-containing membranes and their fusion with the plasma membrane could supply domains with cav-1 and lipids for caveolae formation. Besides it has been described that lipid redistribution in the plasma membrane may modify the biophysical properties of microdomains and regulate signalling pathways [63]. The exact relationship between microdomain composition, density and size is not well established to date. What is known though from previously published data on lipid model systems is that the size of lipid rafts depends on the lipid membrane composition [33], [34] strongly implying that cells could tune their membrane composition to create or destroy domains [64].

A large body of work indicates that cav-1 expression is essential for caveolae formation and function [65], [66]. Moreover, it has been demonstrated in cardiac cells that caveolae integrity regulates α_1A_-AR signalling [67], [68]. It is essential to note that all α_1A_-AR may not necessarily be associated to caveolae. In order to study the importance of the caveolar localisation of α_1A_-AR, we examined its functional role in the presence and absence of cav-1. We demonstrated that agonist stimulation of the α_1A_-AR enhanced resistance to reticular stress-induced apoptosis in DU145 cells through the Bax and the caspase-3 pathway. Importantly, we found that cells underexpressing cav-1 showed no resistance to TG-induced apoptosis, thus implying the necessity of caveolae integrity in this process.

Our results strongly suggest that α_1A_-AR signalling in DU145 cells contributes to their resistance to TG-induced apoptosis *via* caveolae. Additionally, our data add to the growing evidence that cav-1 promotes survival and growth of PCa cells [21], [47], [22]. The elevated expression of α_1A_-AR and cav-1 in DU145 cells may contribute to favouring α_1A_-AR signalling pathway *via* caveolae amplifying the effects on the survival of these cells.

To date, no relevant data describes α_1A_-AR expression variations according to prostate pathological state. Our study was innovative in the investigation of both α_1A_-AR and cav-1 expression in normal, cancerous and BPH samples. We show that the elevated expression of α_1A_-AR in prostate epithelial cells is correlated with prostate pathological progression. During prostate cancer, the organ's architecture is greatly compromised, characterized among others, by proliferation of epithelial cells and invasion of prostate ducts and acini [69]. Our immunohistofluorescent study provides evidence of numerous cytokeratin-18-positive (apical epithelial) cells invading acini in advanced PCa samples highly expressing cav-1 and α_1A_-AR as compared to normal or hyperplastic prostate. Accordingly, we demonstrated increased mRNA levels of cytokeratin-18, α_1A_-AR and cav-1 in androgen-independent PCa samples as compared to BPH samples. In agreement with previously published data our results propose that the role of α_1A_-AR in BPH is related to its presence in the fibromuscular stroma [45], [70], [71]. Further, elevated expression of both cav-1 and α_1A_-AR in advanced stage PCa epithelial cells provide a strong rationale for the understanding of the significance of α_1A_-AR signalling in androgen-independent PCa.

Previous work from our laboratory has demonstrated that TG-induced apoptosis as well as apoptosis resistance induced by EGF in DU145 cells are mechanisms dependent of intracellular Ca^2+^ signalling [72]. We have observed that PHE treatment has no affect on the intracellular Ca^2+^ concentration (data not shown). This observation strongly suggests that resistance to TG-induced apoptosis in DU145 by PHE does not involve the Ca^2+^ signalling pathway. α_1A_-AR signalling is complex and various lines of evidence demonstrate that α_1A_-AR stimulation can activate a variety of signalling pathways depending on cell type, receptor expression and agonist concentration [73], [74]. It has been previously described, in cardiomyocytes, that α_1A_-AR activate ERK-related survival pathways [75]. We have revealed, for the first time, the involvement of the ERK1/2 MAPK pathway in the apoptosis resistance function of the α_1A_-AR in DU145 androgen-independent prostate cancer cells. Interestingly, it has been demonstrated that human prostate biopsies have increased levels of activated ERKs in malignant tissues as compared to those in benign specimens [50], [51].

In conclusion, we demonstrate that caveolae of androgen-independent prostate cancer cells DU145 are involved in the apoptosis resistance induced by the α_1A_-AR. Our data show that caveolae integrity is necessary for α_1A_-AR function in these cells. This study reveals that α_1A_-AR and cav-1 expressions are positively correlated with the pathological state of human prostate and are highly expressed in advance PCa tissue samples. Our data strongly suggest that the protective function of the α_1A_-AR *via* caveolae described in DU145 cells, may contribute in synergy with other factors, to the apoptosis resistance mechanisms of PCa cells and enhancement of their survival.

## Supporting Information

Figure S1 Representative cell cycle profiles of propidium iodide (PI)-stained (A) DU145, (B) DUshCTL and (C) DUshcav-1 cells obtained by flux cytometry as described in the “Methods” section. (a) Non-treated cells, (b) cells treated by 10 µM TG for 48 h, cells pre-treated for three days by 10 µM PHE alone (c), or simultaneously with 1 µM PRA (d), followed by 10 µM TG for 48 h. In all cases, TG treatment induces an increase in the percentage of cells in SubG1 phase, thus apoptotic cells (A, a and b; B, a and b; C, a and b). In PHE pre-treatment conditions in DU145 and DUshCTL cells induced a decrease in the percentage of cells in SubG1 phase as compared to TG alone (A, c; B, c). PHE pre-treatment had no effect on the percentage of DUshcav-1 cells in SubG1 phase (C, b and c). The anti-apoptotic effect of PHE was counteracted by PRA in DU145 (A, d) and DUshCTL cells (B, d) where a higher number of apoptotic cells was observed as compared to pretreatment by PHE alone, but this was not observed in Dushcav-1 cells (C, d). It should be noted that PHE (D, 2) or PRA (D, 3) alone had no effect on the cell cycle phases as compared to control DU145 cells (D, 1) (as well as DushCTL and Dushcav-1).
(0.56 MB PDF)

Figure S2 Representative examples of Hoechst-stained (A) DU145 wild-type, (B) DUshCTL and (C) DUshcav-1 cells. Cells were (a) non-treated, (b) treated by 10ÂµM TG for 48 h, (c) pre-treated by 10ÂµM PHE for 3 days followed by 48 h 10ÂµM TG or (d) pre-treated simultaneously by 10ÂµM PHE and 1ÂµM PRA for 3 days followed by 48 h 10ÂµM TG (as described in the “Methods” section). Cells fixed in ice-cold methanol (15 min) were stained by 4Âµg/ml Hoechst 33528 (Sigma) for 30 min. Stained nuclei were observed at 435 nm using a fluorescence microscope (AxioImager, Zeiss). A total of 500 stained nuclei per condition (three slides per condition) were considered and typical apoptotic figures (chromatin condensation and nuclear fragmentation) were counted, indicated here by the white arrowheads. In (A), (B) and (C) we observed a higher level of apoptosis in TG-treated conditions (b) as compared to (a) non-treated conditions. A three day PHE pre-treatment followed by TG induced a lower number of apoptotic cells (A, c) and (B, c) than TG alone (A, b and B, b). This effect was not observed in DUshcav-1 cells (C) where the number of apoptotic cells was almost the same in b, c and d. The anti-apoptotic effect of PHE was counteracted by PRA in DU145 (A, d) and DUshCTL cells (B, d) where a higher number of apoptotic cells was observed as compared to pretreatment by PHE alone. These results are in agreement with our observation of percentage of apoptotic cells in the SubG1 cell cycle phase (Figure 4A, b).
(2.59 MB TIF)
