# Supplementary figures and images for: Caveolae Contribute to the Apoptosis Resistance Induced by the α1A-Adrenoceptor in Androgen-Independent Prostate Cancer Cells
Source: PLoS One. 2009 Sep 18;4(9):e7068. doi: 10.1371/journal.pone.0007068 (PMC2742726; doi:10.1371/journal.pone.0007068)

# Supplementary data: Figure 1A and 1B

**A**

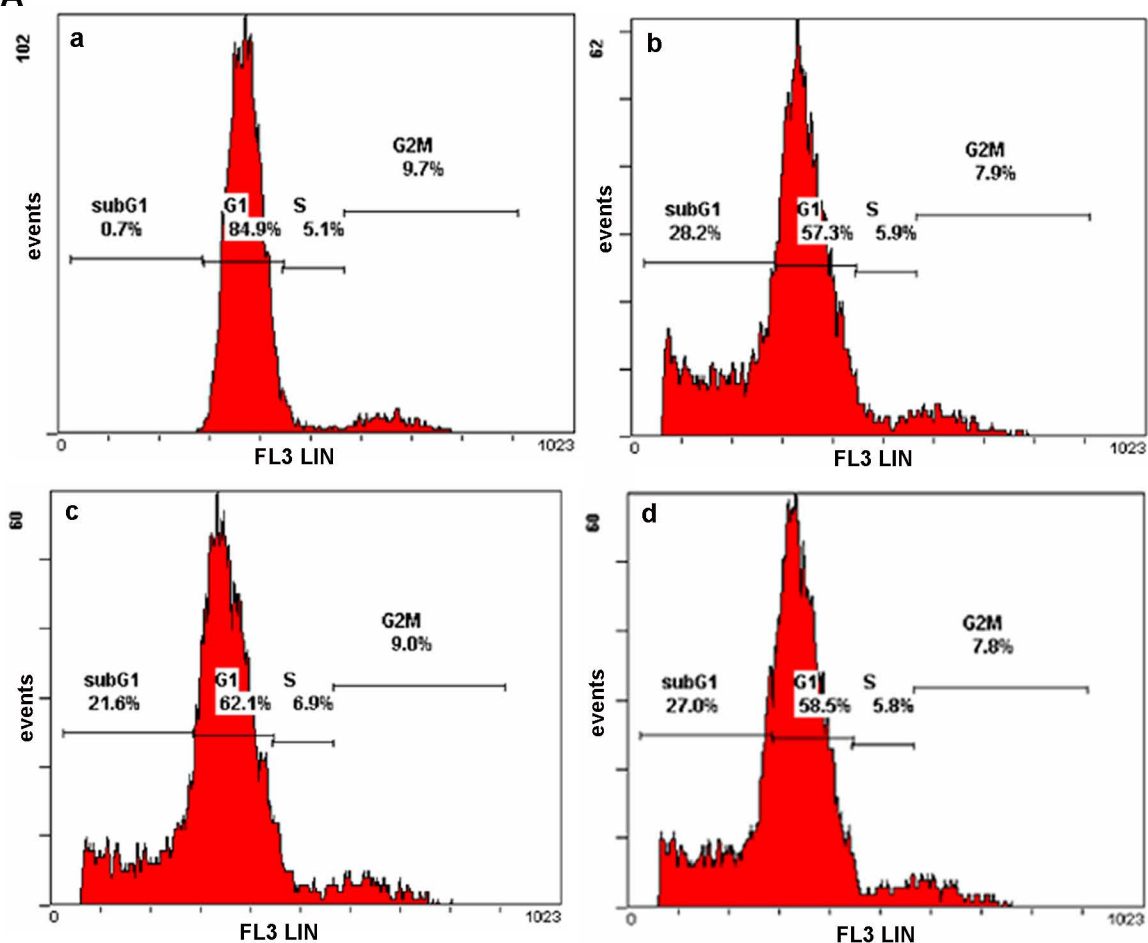

**B**

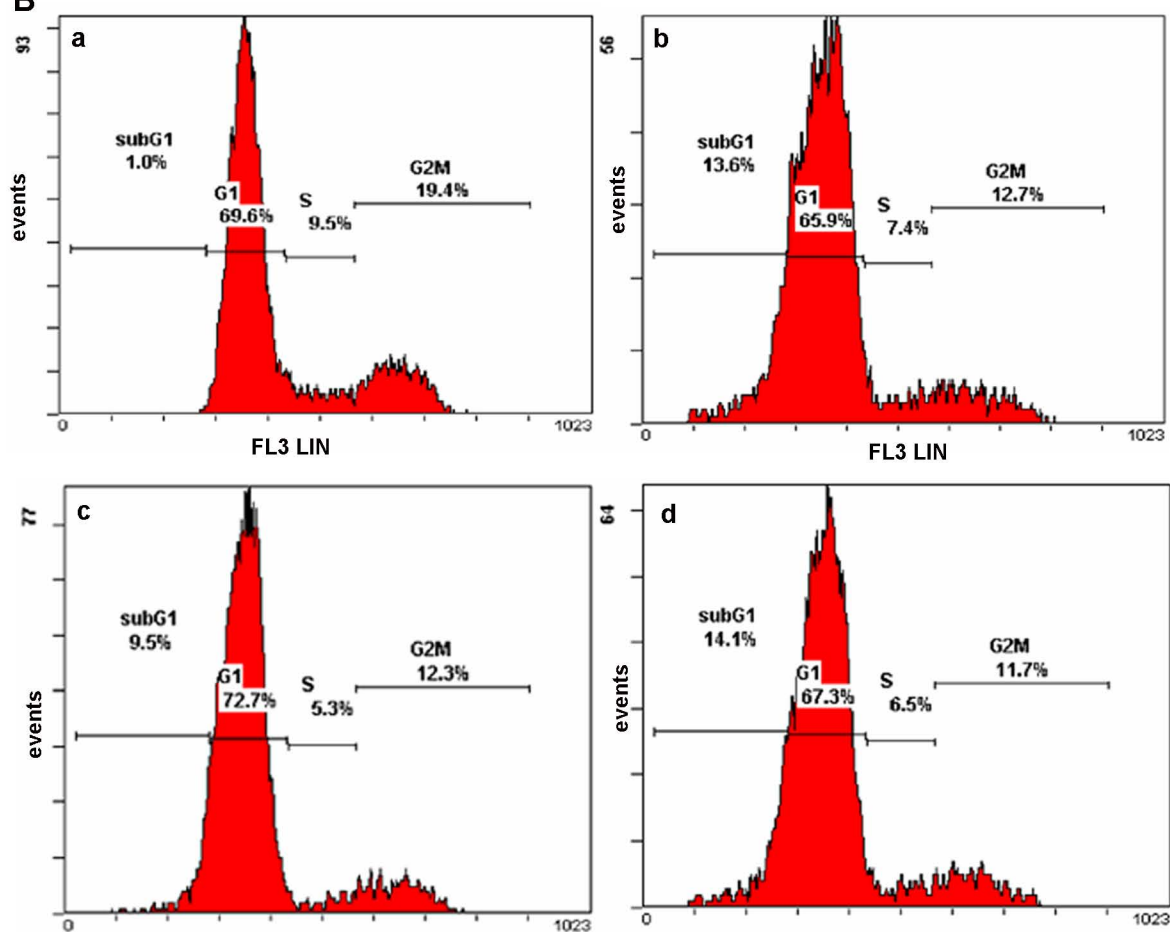

# Supplementary data: Figure 1C and 1D

C

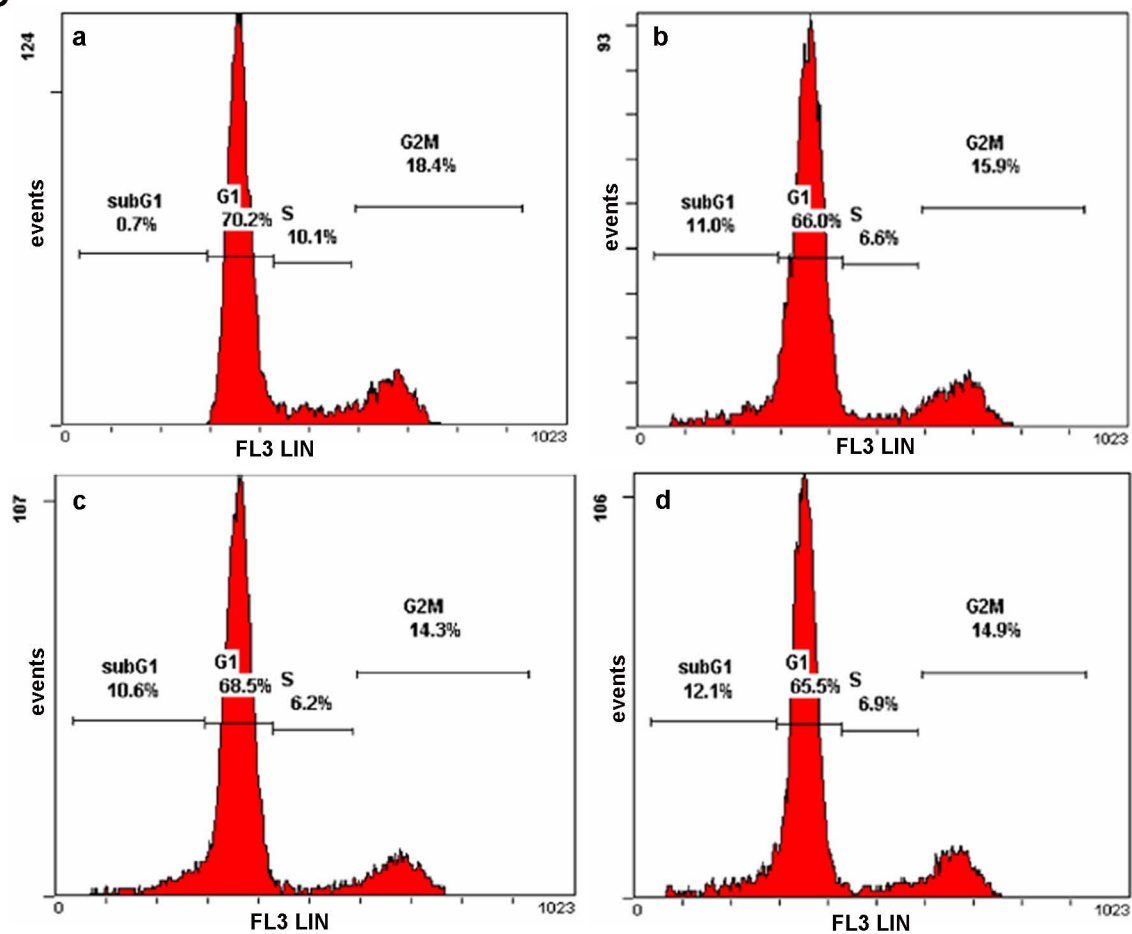

D

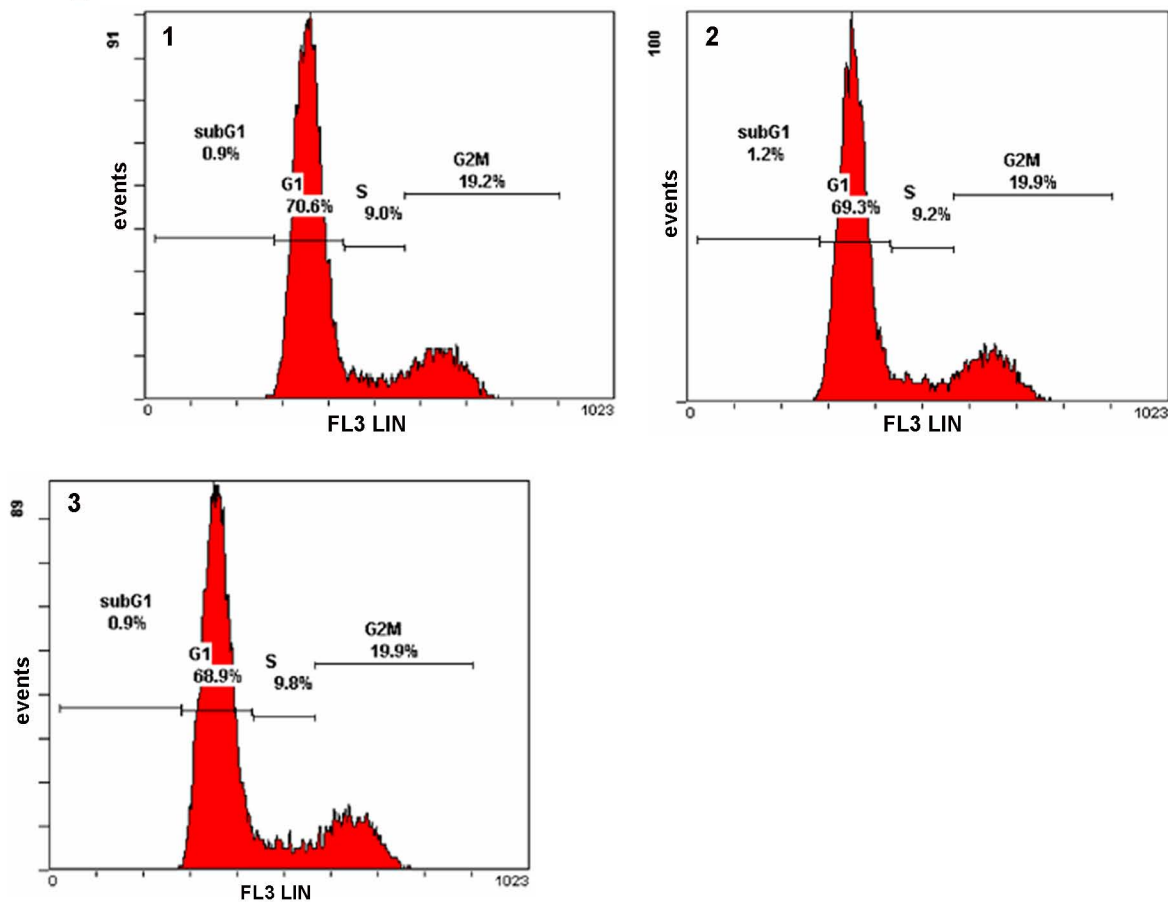

Supplement: Figure S1 — Representative cell cycle profiles of propidium iodide (PI)-stained (A) DU145, (B) DUshCTL and (C) DUshcav-1 cells obtained by flux cytometry as described in the “Methods” section. (a) Non-treated cells, (b) cells treated by 10 µM TG for 48 h, cells pre-treated for three days by 10 µM PHE alone (c), or simultaneously with 1 µM PRA (d), followed by 10 µM TG for 48 h. In all cases, TG treatment induces an increase in the percentage of cells in SubG1 phase, thus apoptotic cells (A, a and b; B, a and b; C, a and b). In PHE pre-treatment conditions in DU145 and DUshCTL cells induced a decrease in the percentage of cells in SubG1 phase as compared to TG alone (A, c; B, c). PHE pre-treatment had no effect on the percentage of DUshcav-1 cells in SubG1 phase (C, b and c). The anti-apoptotic effect of PHE was counteracted by PRA in DU145 (A, d) and DUshCTL cells (B, d) where a higher number of apoptotic cells was observed as compared to pretreatment by PHE alone, but this was not observed in Dushcav-1 cells (C, d). It should be noted that PHE (D, 2) or PRA (D, 3) alone had no effect on the cell cycle phases as compared to control DU145 cells (D, 1) (as well as DushCTL and Dushcav-1). (0.56 MB PDF) [file pone.0007068.s001.pdf]

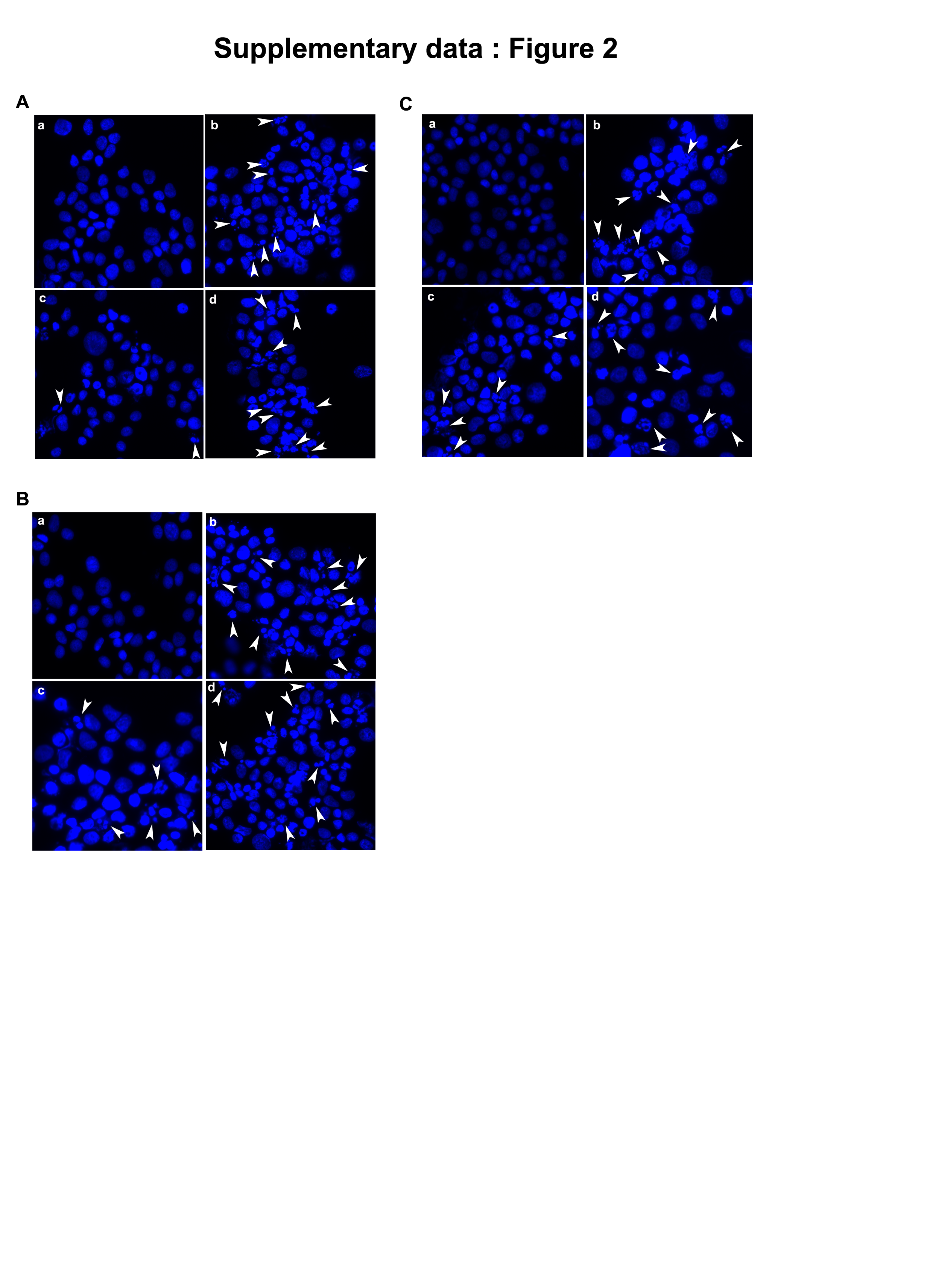

Supplement: Figure S2 — Representative examples of Hoechst-stained (A) DU145 wild-type, (B) DUshCTL and (C) DUshcav-1 cells. Cells were (a) non-treated, (b) treated by 10ÂµM TG for 48 h, (c) pre-treated by 10ÂµM PHE for 3 days followed by 48 h 10ÂµM TG or (d) pre-treated simultaneously by 10ÂµM PHE and 1ÂµM PRA for 3 days followed by 48 h 10ÂµM TG (as described in the “Methods” section). Cells fixed in ice-cold methanol (15 min) were stained by 4Âµg/ml Hoechst 33528 (Sigma) for 30 min. Stained nuclei were observed at 435 nm using a fluorescence microscope (AxioImager, Zeiss). A total of 500 stained nuclei per condition (three slides per condition) were considered and typical apoptotic figures (chromatin condensation and nuclear fragmentation) were counted, indicated here by the white arrowheads. In (A), (B) and (C) we observed a higher level of apoptosis in TG-treated conditions (b) as compared to (a) non-treated conditions. A three day PHE pre-treatment followed by TG induced a lower number of apoptotic cells (A, c) and (B, c) than TG alone (A, b and B, b). This effect was not observed in DUshcav-1 cells (C) where the number of apoptotic cells was almost the same in b, c and d. The anti-apoptotic effect of PHE was counteracted by PRA in DU145 (A, d) and DUshCTL cells (B, d) where a higher number of apoptotic cells was observed as compared to pretreatment by PHE alone. These results are in agreement with our observation of percentage of apoptotic cells in the SubG1 cell cycle phase (Figure 4A, b). (2.59 MB TIF) [file pone.0007068.s002.tif]
